# Distribution patterns of toxic metals in the marine oyster *Saccostrea cucullata* from the Arabian Sea in Oman: spatial, temporal, and size variations

**DOI:** 10.1186/2193-1801-2-282

**Published:** 2013-06-27

**Authors:** Poulose Yesudhason, Moza Al-Busaidi, Waleed AK Al-Rahbi, Aaliah S Al-Waili, Adel K Al-Nakhaili, Nashwa A Al-Mazrooei, Saoud H Al-Habsi

**Affiliations:** Fishery Quality Control Center, Ministry of Agriculture and Fisheries Wealth, 427, 100 Muscat, Sultanate of Oman

## Abstract

The variations in size and spatial and temporal variations in concentrations of toxic metals (cadmium, mercury, and lead) in oyster tissues were studied. Samples were collected at monthly intervals over a 1-year period from three locations along the southern coast of Oman (Mirbat, Hadbeen, and Sadah). Cadmium and lead were analyzed using an inductively coupled plasma atomic emission spectrometer, and mercury was analyzed using a direct mercury analyzer. The annual mean concentrations in oyster tissues sampled from the three locations and from different time periods ranged from 2.64 to 3.80 mg kg^-1^ for cadmium, 0.009 to 0.02 mg kg^-1^ for lead, and 0.01 to 0.02 mg kg^-1^ for mercury. The temporal effect on cadmium concentrations was more distinct than the local site-specific effect, with higher concentrations recorded in tissues during the summer season than in the winter season. Moreover, within each site, a significant time-specific dependence on the toxic metal concentration differences was recorded. Lipid content was found to influence mercury concentrations in the oysters; however, there was no relationship between cadmium or lead and moisture or lipid content. No distinct relationships were observed between the size of oysters and metal uptake by the oyster. The results were discussed in relation to those obtained from related species in the seas of Oman and worldwide.

## Introduction

Mercury, cadmium, and lead are the major toxic metals that cause environmental degradation in marine ecosystems (Matta et al. 1999). Toxic metals are introduced into the marine environment by natural and anthropogenic activities (O’Conner 1996). These metals accumulate in the bodies of marine biota at concentrations much higher than those found in the ambient water and are biomagnified in the food chain at higher trophic levels, posing a risk to human consumers (Saka et al. 2006). Monitoring programs and research of metal concentrations in environmental samples are being widely conducted because of concerns over accumulation and toxic effects (Otchere 2003). Bivalves are widely used as indicators to assess the bioavailability of metals in coastal waters in many parts of the world (Blackmore 2001; Cohen et al. 2001; Sunlu 2002), because of their ability to accumulate heavy metals in their tissues at concentrations many times higher than those in the surrounding water. Therefore, contaminant concentrations in the tissues of bivalves more accurately reflect the magnitude of environmental contamination (Phillips 1990). Heavy metal concentrations and accumulation in these organisms are influenced by several factors, including metal bioavailability, sampling season, hydrodynamics of the environment, size, sex, changes in tissue composition and reproductive cycle of the selected organism, salinity, organic matter, food acquisition capability, and size-weight relationships (Boyden and Phillips 1981). Seasonal variations in metal concentrations have been related, to a great extent, to seasonal changes in flesh weight during the development of gonadal tissues (Joiris et al. 2000). Metal concentrations in bivalves at the same location differ between different species and individuals because of a species-specific ability to regulate or accumulate trace metals (Otchere et al. 2003). Different animals in the same community at the same trophic level may accumulate pollutants differently because of differences in the physical and chemical properties of the habitat (Chouvelon et al. 2009).

Oysters have already been proposed as a biomonitor for marine ecological tests to assess the water quality because of their sensitive nature and rapid response to pollutants (Szefer 2002; Phillips 1990). *Saccostrea cucullata* is the most abundant bivalve in Oman and is normally found attached to rocks. Studies conducted by de Mora et al. (2004) and Fowler et al. (1993) on bivalves have shown significant amounts of metals in the soft tissues of oysters in these regions. However, relatively low levels of industrial activities are found in this area. The concentrations of metals in tissues that can be attributed to environmental exposure remain ambiguous because of various biological and ecological factors that affect the accumulation and concentration of toxic metals in oyster tissues. There is an increasing necessity to control the quality of coastal waters and marine biota that may be influenced by different anthropogenic inputs into the marine environment. In this study, we attempted to understand the real situation of toxic metal accumulation in edible oyster tissues in Oman by using a 1-year dataset.

Although a number of papers have been published on heavy metal levels observed in fish, shellfish, and sediments (Al-Hashami et al. 1991; Al-Sayed et al. 1994; Fowler et al. 1993; de Mora et al. 2004; Al-Busaidi et al. 2011) in the Persian Gulf and the Gulf of Oman’s environment, to our knowledge, the present study is the first to detail heavy metal bioaccumulation and temporal variation in bivalve species from these regions. The main objectives of the present study are to investigate metal contamination in the rock oyster at different sampling sites with varying degrees of urbanization in the coastal waters of the Dhofar Governorate, Oman, and to elucidate the temporal changes and size dependence of metal concentrations in oyster tissues. The knowledge of heavy metal concentrations in native species is very important with respect to natural resource management, human consumption, public health, and identification of the most polluted areas.

## Materials and methods

### Study area

The Dhofar Governorate lies in southern Oman, on the eastern border with Yemen. Its mountainous area covers 99,300 km^2^ and has a population of 215,960. It lies between 18°0′0′′N and 54°0′0′′E. Dhofar and a small portion of the northern tip of Yemen are directly exposed to the southwest monsoon, known as the Khareef, from mid-August to late September or early October. The coast of Dhofar is fertile, since it is watered by monsoonal fogs from the Indian Ocean, and is a part of the Arabian Peninsula coastal fog desert eco region. The Dhofar Governorate has agricultural lands and industrial plants (iron-steel plants, beverage, LPG plants, oil transfer docks, and other industrial plants); boating activity is also prevalent in the governorate. Mirbat (A), Hadbeen (B), and Sadah (C) were the 3 locations selected for oyster sampling in the Dhofar Governorate. Mirbat is located at approximately 16°59′19″N and 54°41′32″E. Hadbeen (17°14′54′′ N, 55°13′41″E) is famous for its lobsters and abalone, as well as for several other varieties of fish. Sadah (17°04′15′′N, 55°06′E) is situated just shoreward of a major shipping lane for tanker traffic passing through the Strait of Hormuz and is thus vulnerable to the potential effects of contaminants originating from accidents or intentional releases. Rock oysters are abundant along the coast of the entire Dhofar Governorate and have an important role in the food web of marine ecosystems. These areas were selected based on the unique aspects and sensitive nature of their marine ecology and results from previous literature on metal accumulation.

### Sample collection

**Figure 1:**
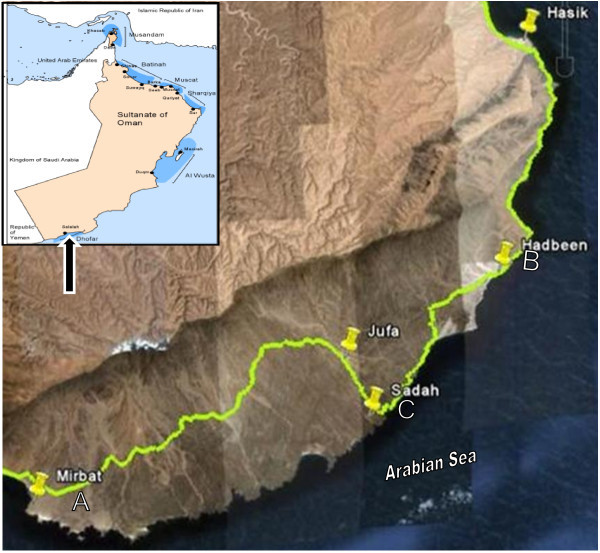
**MAP of study area showing the location of sampling sites (Mirbat, Sadah and Hadbeen).A:** Mirbat, **B:** Sadah and **C:** Hadbeen.

The 3 sampling stations, Mirbat (A), Hadbeen (B) and Sadah (C), selected for the presence of rock oyster along the coastline of Dhofar in the Arabian Sea of Oman are presented in the Figure 1. One oyster bed was chosen at each of the 3 locations, and oysters were collected over a 12 month period from June 2009 to May 2010. Approximately 150 individuals of rock oyster of different shell length were manually collected from depths of 0–4 m from each station at a single sampling point each month. Sampling was performed on the same day at all 3 stations to avoid possible variations in metal content due to weather conditions. After all samples were removed, and they were washed with clean seawater at the point of collection, placed into clean plastic bags, and transferred in a cool box to the laboratory. Samples were frozen at −20°C until sample preparation.

### Sample preparation

For metal analysis, after thawing, the samples were divided into small (20–40 cm), medium (40–60 cm) and large (>60 cm) size classes based on shell length, and the total weight of each individual was measured to the nearest 0.1 g. The shell length (SL, the longest shell dimension) of each individual oyster was determined to the nearest 0.1 mm using Vernier calipers. The whole oysters were dissected on a clean bench with the aid of a stainless steel knife which had been cleaned with acetone and hot distilled water prior to use. All samples were packed separately in polyethylene bags with proper labeling and used in subsequent analyses.

### Chemicals

All reagents were analytical reagent grade and milliQ 18 mΩ pure water (MilliQ, Millipore, USA) was used for preparation of reagents and standards. Calibration standards of mercury, lead and cadmium (1000 mg L^-1^ in 2% HNO_3_) were purchased from Merck, Germany. The standard solutions for calibration were prepared from a stock solution of 1000 mg L^-1^ by successive dilutions with 1.5% nitric acid. The standards were diluted appropriately and used to calibrate the Inductively Coupled Plasma Atomic Emission Spectrometer (ICP OES) and the Direct Mercury Analyzer (DMA). All glassware and other containers were thoroughly cleaned with 1.5% (w/v) nitric acid solution then rinsed with MilliQ water several times and air-dried prior to use.

### Lipid and moisture analyses

Lipid content (%) of the oyster tissue was determined using the Soxhlet method (Buchi Extraction System B-811) using chloroform/methanol (2:1) as extract solvents. In addition, the moisture content was calculated as the weight loss of the heated sample at 110°C.

### Heavy metals analysis

Metal analyses were performed by the method of Uysal et al. (2008) with some modifications. For each sample, 0.4 g of homogenized oyster tissue (wet weight) was accurately weighed and transferred into a 25 mL quartz vessel together with 5 mL of concentrated (65%) nitric acid (HNO_3_) and 1 mL of (35%) hydrogen peroxide (H_2_O_2_)_._ The extraction vessel was placed in a microwave digestion system (Milestone ETHOS PLUS, Italy), and the digestion program was run in 2 steps (step (1) 25–180°C for 30 min at 1000 W; step (2) 180°C for 15 min at 1000 W). All the digested samples were diluted to 25 mL with milliQ water in pre-acid washed standard flasks and used for analysis. Cadmium and lead were analyzed using an Inductively Coupled Plasma Atomic Emission Spectrometer (ICP AES, ICPE-9000, Shimadzu, Japan) at 220 nm for cadmium and 210 nm for lead. A standard torch for this instrument was used with an outer argon gas flow rate of 15 L min^-1^ and an intermediate gas flow of 0.9 L min^-1^. The applied power was 1.0 kW. For mercury, 0.1 g of oyster soft tissue was accurately weighed in a sample boat, and total mercury was determined using a Direct Mercury Analyzer (DMA-80, Milestone, Italy) without further sample preparation. Typical repeatability was 3.2-4.4% for cadmium, 2.4–4.7% for lead and 2.5–3.8% for mercury. Good reproducibility of the method was obtained, yielding the intra-day and inter-day relative standard deviations less than 5%. The instrument detection limits were 0.01 mg kg^-1^ for cadmium 0.02 mg kg^-1^ for lead and 0.5 ng g^-1^ for mercury.

### Quality control

All samples were taken in triplicate and all measurements were run in triplicate for standards and samples. Metal concentrations were calculated and expressed in mg kg^-1^ wet weight. The analytical blanks were run in the same way as the samples and concentrations were determined using standard solutions prepared in the same acid matrix. Accuracy of the analytical method was monitored by analyzing certified reference material (Mussel tissue - NIST SRM 2976). The standard reference material was analyzed during each batch (n = 20) and the heavy metal recovery rates were 85% for cadmium, 92% for mercury and 90% for lead. A recovery test was carried out by spiking standard solutions of heavy metals in homogenized samples. The recoveries for heavy metals during these experiments were found to be between 80-110%; no batches were outside these limits.

### Statistical analysis

Results of heavy metal concentrations were analyzed using SPSS 19.0 (SPSS INC., Chicago, IL) software. One-way and two-way ANOVA, Duncan’s multiple range and Pearson correlation coefficients were used to examine differences between spatial, temporal and size class observations and metal concentrations. A significance level of 95% was used.

## Results and discussion

### Annual mean metal concentration

The calculated annual mean concentrations of mercury, cadmium and lead in rock oyster collected from the Dhofar coastal areas in the Arabian Sea from June 2009 to May 2010 were 0.017 ± 0.008 mg kg^-1^, 3.31 ± 1.14 mg kg^-1^, and 0.011 ± 0.03 mg kg^-1^, respectively. The presence of these metals in the rock oysters reflects the bioavailability for incorporation by oysters along the Dhofar coast of the Arabian Sea, Oman. It has been well documented that the rock oyster, *S. cucullata*, is capable of bioaccumulating toxic metals in its soft tissues and metal concentrations within this study were, on average, within the variations commonly described in the literature for natural areas or areas slightly affected by metal pollution (Table 1). Based on the maximum permissible levels set by the Omani regulation and the European Union, the metal concentrations in the oysters sampled at the 3 sites along the Dhofar coastal area were within the legal standards for mercury and lead (1.5 mg kg^-1^ wet weight). Cadmium concentrations in *S. cucullata* exceeded the maximum limits allowed for human consumption (1 mg kg^-1^ wet weight) under EU (EC, 2001) and Omani legislation (Ministerial Decision No.12/2009 2009).

**Table 1:** **Comparison of metals concentrations in*S.cucullata*from different parts of the world**

Location	Cd (mg kg^-1^)	Pb (mg kg^-1^)	Hg (mg kg^-1^)	Reference
Australia	0.14-4.07	0-0.46		Peerzada et al.,^1990^
	0.29-10.63	2.59-9.38		Peerzada et al.,^1992^
	0.17-9.1			Peerzada et al.,^1993^
Hongkong	2.07- 4.05			Blackmore 2001
India	1.82 ± 0.4	0.308 ± 0.07		Kumar et al., 1990
	(1.25-3.19)	(0.20-2.02)		
UAE	1.53	0.0625	0.007	de Mora et al., 2004
China	2.43 ±1.37	0.120 ± 0.044	0.027 ±0.011	Fang et al., 2004
	(1.27-4.86)	(0.051-0.168)	(0.013-0.044)	
Oman	0.7-8.77	0.015-0.55	-	Fowler et al., 1993
Oman	1.55-4.825	0.067-0.95	-	Fowler 1988
Oman	2.24-5.47	0.096-0.168	0.012-0.038	de Mora et al., 2004
	(3.55)	(0.127)	(0.028)	
Al-sawadi	4.975	0.168	0.036	
Ras AlHamra	2.457	0.137	0.038	
Ras Al-yei	5.475	0.096	0.012	
Hilf	2.65	0.111	0.038	
Mirbat	**2.24**	**0.125**	**0.019**	
**Oman**	**3.31 ± 1.14**	**0.0119 ± 0.031**	**0.017 ± 0.008**	**This study**
	**(1.58-6.2)**	**(ND-0.129)**	**(0.008-049)**	
Mirbat	**2.64 ± 1.19**	**0.009 ± 0.01**	**0.015 ± 0.006**	
Hadbeen	**3.48 ± 0.80**	**0.009 ± 0.023**	**0.013 ± 0.006**	
Sadah	**3.80 ± 1.14**	**0.023 ± 0.048**	**0.022 ± 0.010**	

- : not analyzed; number in parenthesis indicates the range.

### Spatial distribution

**Figure 2:**
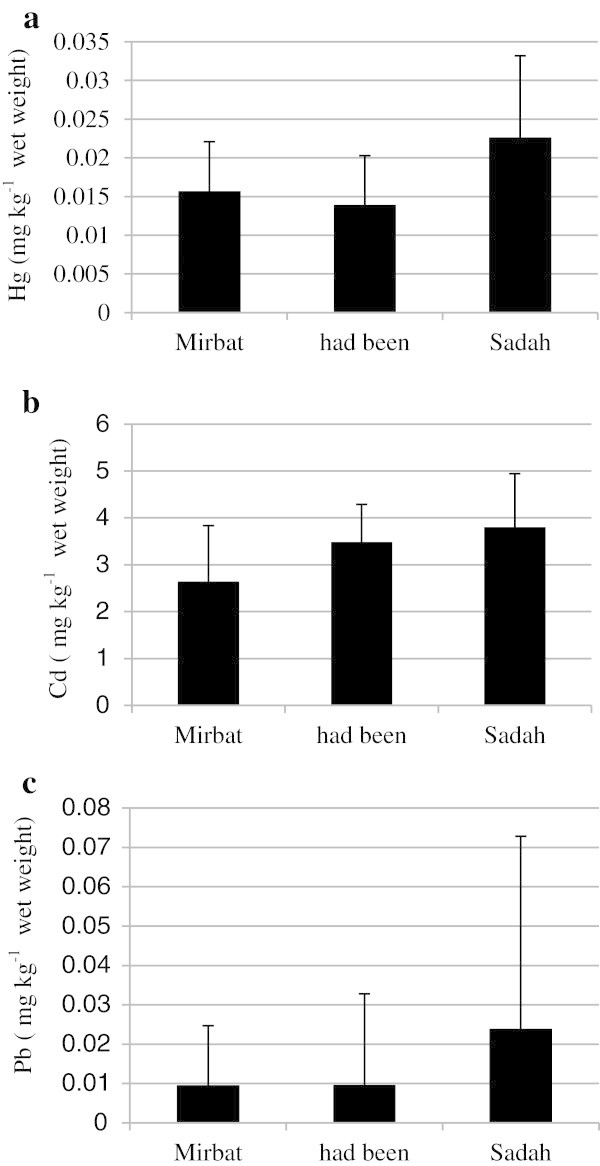
**Spatial distribution of mercury (a), cadmium (b) and lead (c) concentrations (mean ± SD) in soft tissue of rock oyster*S. cucullata.***

Patterns in the spatial distribution of toxic metals measured in the soft tissues of oysters across the study sites are shown in Figures 2a-c. Mercury concentrations in the tissues of oysters ranged from 0.01-0.03 mg kg^-1^ in Mirbat, 0.008-0.03 mg kg^-1^ in Hadbeen and 0.009-0.05 mg kg^-1^ in Sadah. Cadmium concentrations varied from 1.58-5.74 mg kg^-1^ in Mirbat, 2.52-4.97 mg kg^-1^ in Hadbeen and 2.23-6.2 mg kg^-1^ in Sadah. In the case of lead, concentrations in the oysters were quite low and below detection limits for the majority of the time, ranging from 0.001-0.02 mg kg^-1^ in Mirbat, 0.04-0.07 mg kg^-1^ in Hadbeen and 0.03-0.13 mg kg^-1^ in Sadah. Relatively higher concentrations of mercury were found in oysters from Sadah compared to oysters from the remaining 2 sites, which had comparable values. However, the concentrations of mercury at the other 2 sites do not reflect additional input, only natural variations.

Cadmium concentrations were significantly higher in oysters from Sadah than from Mirbat; however, no significant differences in the accumulation pattern were observed between Sadah and Hadbeen (Figure 2b). Higher cadmium values at Sadah, which is relatively close to Mirbat where lower concentrations were observed, reinforce results found by Lima (1997) who reported a wide range of concentrations of this metal in individuals from the same population. On the other hand, variation in cadmium concentrations may be due to environmental variables, which possibly influence the bioaccumulation of this metal. de Mora et al. (2005) observed the same pattern when determining cadmium concentrations in marine benthic algae in this region. Human activities and boat activities are lower in the area surrounding Mirbat compared with the other 2 study sites; thus, metals from anthropogenic sources are present at lower concentrations within the environment, and their availability for uptake by oysters is minimal at this study site.

In contrast to the trend for cadmium, lead was present in very low concentrations in oysters throughout the year at all 3 study sites (Figure 2c). As was observed for cadmium, the lowest and highest concentrations of lead were detected in oysters from Mirbat (0.002 mg kg^-1^) and Sadah (0.02 mg kg^-1^), respectively. However, no significant differences (p > 0.05) were found in the concentrations of lead in oysters from the 3 sampling sites, suggesting similar bioavailability of this metal at all sites. Higher concentrations of lead in oysters sampled from Sadah compared to oysters from Mirbat and Hadbeen may be related to anthropogenic inputs at Sadah, although concentrations of lead recorded were low. Trefry et al. (1995) reported that the concentration of lead in tissues depends mainly on its concentration in seawater and the creation of a compound with dissolved organic matter. Phillips et al. (1982) and Riget et al. (1997) reported that lead was not significantly accumulated by marine biota, even when its concentration was high in abiotic compartments. Besides, the main source of lead in the marine, atmospheric deposition has been drastically reduced following the prohibition of this metal in gasoline and the introduction of unleaded gasoline, which was observed during this study (Table 1). Paes-Osuna et al. (2002) observed a significant correlation between the concentration of lead and polycyclic aromatic hydrocarbons in oysters collected in Mexico, suggesting that the source of both pollutants may be the local small fishing boats, which are also common in Sadah.

### Temporal variations

**Figure 3:**
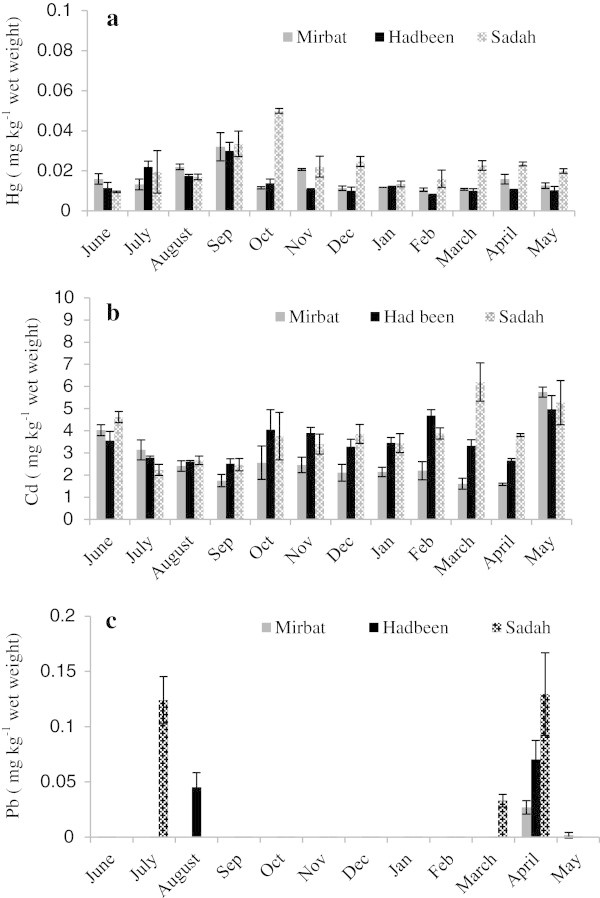
**Temporal distributions of mercury (a), cadmium (b) and lead (c) concentrations (mean ± SD) in the soft tissue of rock oyster*S. cucullata.***

There were significant temporal variations (p < 0.05) in mercury, cadmium and lead concentrations in oysters from the Dhofar coast of the Arabian Sea (Figure 3a-c). According to Haynes and Tooley (Haynes and Toohey 1998), seasonal differences in metal concentrations in mussels from non-impacted environments are usually related to physio-chemical variations in the water, which can alter metal bioavailability and/or the feeding rates of the organisms. Bryan (1993) mentioned that temporal variations in metal concentrations in bivalves are related to variations in local phytoplankton productivity. His observations are reinforced by the fact that an increase in phytoplankton productivity during upwelling results in an increase in a bivalve’s nutritional status, which in turn leads to an increase in metal concentrations in the organism.

Temporal variations in mercury concentrations during the sampling period were statistically significant (p < 0.05) in oysters from the study sites. At Mirbat, the highest mercury concentration of 0.03 mg kg^-1^ was recorded in September 2009 and the lowest of 0.01 mg kg^-1^ in February 2010 (Figure 3a). Like oysters from Mirbat, mercury concentrations in the tissues of oysters from Hadbeen ranged between a low of 0.008 mg kg^-1^ in February 2010 and a high of 0.03 mg kg^-1^ in September 2009. At Sadah, total mercury concentrations for the oysters ranged from a low of 0.009 mg kg^-1^ in June to a high of 0.05 mg kg^-1^ in October 2009 (Figure 3a). Oysters exhibited a gradual rise in mercury concentrations from June to September 2009, coinciding with the onset of the spawning season for oysters. There was, however, a declining trend or no significantly change (p > 0.05) in mercury concentrations from November 2009 to May 2010, except in oysters from Sadah where an increasing trend was observed from January 2010 to April 2010. Temporal variation in the concentration of mercury in oysters from Sadah followed a non-uniform trend in October recording a peak value of 0.05 mg kg^-1^, which correlated well with the lipid content obtained during the same month. The concentration of mercury in oysters at Sadah was 3 times higher than the general concentration level obtained in oysters from the other 2 study sites. A similar time accumulation pattern was observed in Mirbat and Hadbeen, with the highest and lowest mercury concentrations observed in oysters in September 2009 and February 2010, respectively, in this case the profile did not reveal any external sources, thus, we assume these variations are due to natural variations. In Mirbat, mercury concentrations varied through time in a different manner to the variations observed in Sadah and Hadbeen. These results highlight the complexity of the dynamics of heavy metals in the marine environment. Temporal variability could be caused by physiological factors and also natural processes, such as weathering, hydrological conditions, intense leaching of mineralized rock or run-off, particulate matter resuspension and primary production. These processes are highly variable on a periodic basis and could possibly account for the differences in monthly metal concentrations in the oysters.

In the case of cadmium, significant temporal variations (p < 0.05) were observed in concentrations in oyster tissues at all 3 sites (Figure 3b). At Mirbat, the concentrations observed in the tissues of oysters varied from 1.61 mg kg^-1^ in March 2010 to 5.47 mg kg^-1^ in May 2010; at Hadbeen they ranged from 2.52 mg kg^-1^ in September 2009 to 4.97 mg kg^-1^ in May 2010; and at Sadah they varied from 2.23 mg kg^-1^ in July (2009) to 6.2 mg kg^-1^ (March 2010). In May 2010, oysters from all sites exhibited a peak in cadmium concentration, from a value of 1.58 to 5.7 mg kg-^1^ in Mirbat, from 2.66 to 4.9 mg kg-^1^ in Hadbeen, and from 3.8 to 5.2 mg kg-^1^ in Sadah, indicating a common origin of this metal to all 3 study sites. This was probably due to the southwest monsoon winds and associated upwelling in the western Arabian Sea. In the northwest Arabian Sea, upwelling occurs each summer driven by the strong southwest monsoon winds. The upwelling results in high biological productivity and a distinct assemblage of plankton species in the surface waters of Oman that are preserved in the sediments along the Oman continental margin (Anderson and Prell 1993). The strongest monsoon winds (indicated by increased upwelling) occurred during May and gradually decreased in August (upwelling reduced). The intense upwelling and the southwest monsoons were the major sources of high cadmium concentrations observed in the tissues of oysters during May (intense upwelling) and also influenced the onset of the substantial decrease in concentrations observed in September, when upwelling is less intense and other physical mechanisms become important. Shi et al. (2000) reported the persistence of cold upwelling waters for nearly a month after the end of the southwest monsoon along the Oman coast. Plankton cell concentration would provide better and more reliable information on the changes occurring in the Arabian Sea due to the monsoon system. Cadmium concentrations in the tissues of oysters from Mirbat and Hadbeen showed a similar trend to those exhibited in the tissues of oysters from the Sadah region. Our results provide evidence that both the intensity of the monsoon and the associated upwelling are important variables that contribute to the higher cadmium concentrations observed in the tissues of oysters during summer. In addition to natural and anthropogenic inputs, biological variables of oysters, such as size, sex, changes in tissue composition and stage of reproductive cycle, as well as the season of sampling and the hydrodynamics of sea water have to be considered as factors that may influence variations in metal concentrations Madkour (Madkour et al. 2011). Oliver et al. (2002) observed that temporal variation in cadmium concentrations in *Saccostrea commercialis* was correlated with the dry weight of the individuals. The authors observed that cadmium concentrations tended to diminish with increasing dry weight of the individuals, thus, a dilution effect occurs as the organisms grow. A similar pattern was observed during this study.

Concentrations of lead in the tissues of oysters remained less than the detection limit throughout the sampling period, except in April 2010 at all the sampling sites (Figure 3c), in August 2009 at Hadbeen and in March and July at Sadah, indicating significant temporal variations in lead concentrations.

### Relationship between metals and proximate composition

**Figure 4:**
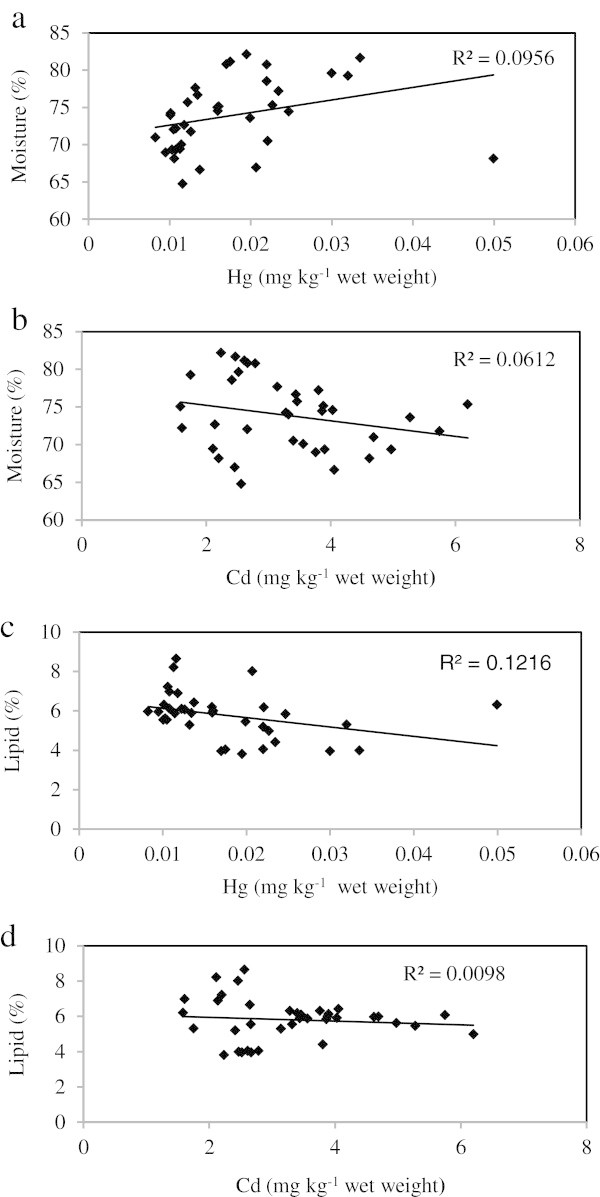
**Multiple regression between metal concentrations and moisture and lipid.**

The relationship between the metal concentrations and proximate composition (lipid and moisture content) of the oysters are illustrated in Figure 4(a-d). Lead was below the detection limit for the majority of the time and so correlations between lead and chemical parameters were not assessed. Moisture content of the oysters ranged from (w/wt) 64.7-79.2% at Mirbat, 66.6-81.1% at Hadbeen and 68.1-82.1% at Sadah. The lowest (October at all the 3 sites) and highest (September: Mirbat, July: Hadbeen and August: Sadah) values of moisture content coincided with post spawning and peaks of spawning, respectively (Sukumar and Joseph 1988). Lipid content of the oysters ranged from 5.1-8.6% at Mirbat, 3.95-6.4% at Hadbeen and 3.8-6.3% at Sadah. In contrast to moisture, the lowest (August: Mirbat; September: Hadbeen; and July: Sadah), and highest (October at all the 3 sites) values of lipid content were reported during spawning (June-September) and post spawning, respectively. Braley (1982) recorded spawning periodicities in *Saccostrea* spp. inhabiting different regions. The correlation between metal concentrations and proximate composition was low at all the study sites except Hadbeen, where the Pearson correlation values were greater than those obtained for Mirbat and Sadah. Differences in mercury and cadmium concentrations were observed in relation to biochemical parameters. The combined r^2^ value indicated a greater correlation between mercury concentrations and lipid content compared to mercury concentrations and moisture content. However, no relationship was observed between cadmium concentrations and moisture or lipid content indicating that the accumulation pattern of this element is less influenced by the biological cycle of the marine oyster *S. cucullata*

### Size effects

**Figure 5:**
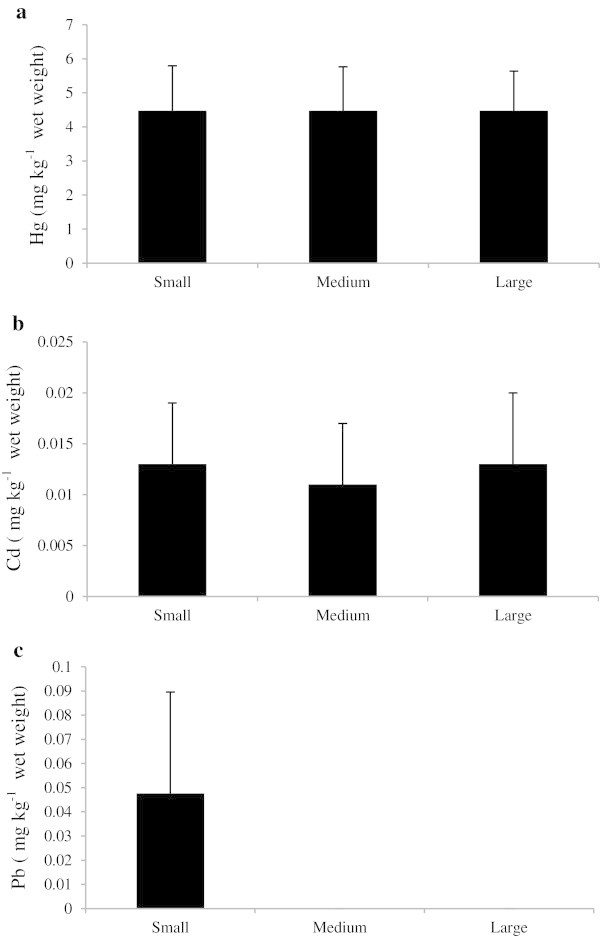
**Toxic metals mercury (a), cadmium (b) and lead (c) in relation to oyster sizes (mean ± SD).**

Generally, oyster samples are harvested and consumed regardless of their size. During the current study, oysters sampled ranged in size from a minimum of approximately 20 mm up to 60 mm. Mercury concentrations were lower in the medium sized oysters in comparison to the small and large sized oysters (Figure 5a). However, medium sized oysters showed relatively higher cadmium concentrations than small and large sized oysters, but this difference was not statistically significant (Figure 5b). Lead was not detected in medium and large sized oysters (Figure 5c). Multiple comparison tests performed after two-way ANOVA on the mean concentrations of metals and the size parameters of oyster tissue (all sampling locations together) indicated no distinct relationship between size and metal uptake by the rock oysters. Large sized oysters contained concentrations of mercury similar to levels observed in small and medium sized oysters, suggesting similar regulating mechanisms of mercury in the tissues of oysters regardless of size. The high cadmium concentration of 6.1 mg kg^-1^ observed in small sized oysters during the months of September and October, in contrast to the relatively low concentrations observed in medium and large oysters, suggests that oysters in the medium and large size classes are sexually mature and have an efficient metabolism and detoxifying process (Connell et al. 1999) to keep the concentration of metals relatively low. In contrast, Ferreira et al. (2005) reported that the prior selection of the size of the individuals in the effect of size on heavy metal accumulations. However, individuals of the same size are not necessarily the same age and weight because of the irregular shape of the substrate to which they are attached; individuals can grow along a different axis from the longitudinal growing pattern expected. Oysters can assume the internal shape of the shell and with enough space to develop their true size is not obvious from their external size. This pattern was observed in oysters from Dhofar, where the substrate was formed by irregular granite rocks.

### Comparisons with previous work

The results of the present study are compared to others in Table 1. According to the results, mean mercury, cadmium and lead concentrations in the soft tissues of *S. cucullata* found during this study fell within the range of concentrations reported in previous studies. Also, we compared our data with an earlier study conducted by de Mora et al. (2004) in the same study area for *S. cucullata* to find inter annual variability in metal concentrations. No significant changes in metal concentrations were observed between both the study areas, except lead (Table 1). A decrease in lead content between years in the same study area can possibly be explained by the increase in use of non-leaded gasoline and fishing equipment in these regions. Although mercury and lead concentrations do not seem to be problematic in terms of human consumption, caution should be taken in regard to their consumption because despite being low, cadmium concentrations in their soft tissue are considerable.

## Conclusions

This study demonstrated that *S. cucullata* accumulates different heavy metals in body tissues at different concentrations. In general, cadmium showed the highest accumulation in oysters followed by mercury and lead. We observed significant spatial variation in the concentrations of metals in *S. cucullata* between the study sites. Regarding temporal variations, increases in cadmium concentrations were observed in samples collected in May 2010, a rainy period, suggesting similar origins of these metals, i.e., the southwest monsoon and upwelling affected all 3 study sites. No distinct relationships were found between metal accumulation and different size classes of oysters. Regular and more detailed monitoring studies, covering more than 1 species at all the study sites, are recommended for the selection of an indicator species.
